# The optimal dosage of pefcitinib for the treatment of active rheumatoid arthritis: A protocol for an updated network meta-analysis

**DOI:** 10.1097/MD.0000000000024586

**Published:** 2021-02-19

**Authors:** Yuyi Zhou, Chunfang Sun, Chunyan Chen

**Affiliations:** Department of Rheumatology and Immunology, Anji Branch of the First Affiliated Hospital of Zhejiang University, Anji, China.

**Keywords:** Janus kinases, network meta-analysis, peficitinib, rheumatoid arthritis, systematic review

## Abstract

**Background:**

Previous meta-analyses have indicated that peficitinib was the promising agent for the treatment of rheumatoid arthritis (RA). Meanwhile, a recent network meta-analysis has further investigated the comparative efficacy of different peficitinib regimes. However, pooled results from previous network meta-analysis must be cautiously interpreted because 2 eligible studies were missed. Therefore, we designed this updated network meta-analysis to further establish the optimal dosage of peficitinib in treating RA.

**Methods:**

We will carry out a network meta-analysis of randomized controlled trials (RCTs) with Markov Chain Monte Carlo method in order to merge direct and indirect evidence. We will identify potentially eligible studies through searching 4 databases including PubMed, Embase, Cochrane Library, and China National Knowledgement Infrastructure (CNKI) until to December 2020. We will make this network meta-analysis following the process recommended by the Cochrane Handbook.

**Discussion:**

As a systematic and chronic autoimmune disease, RA primarily was characterized by persistent synovitis, progressive joint injury, and deformity. Patients who were identified as RA will experience a series of adverse consequences such as disability and poor quality of life (QoL). Peficitinib, one of the Janus kinases (JAKs) inhibitors, has been suggested to be effective in treating active RA by numerous clinical studies and meta-analyses. Although a recent meta-analysis investigated the comparative efficacy of different dosages of peficitinib, reliable results cannot be obtained because it missed 2 critical eligible studies. We designed this updated network meta-analysis through including all eligible studies to further ask which dosages may be the preferred option for treating active RA.

**Ethics and dissemination:**

No ethics approval and informed consent will be required in our meta-analysis. Our findings in this updated network meta-analysis will be disseminated via conferences and academic journal.

**Open Science Framework (OSF) Registration DOI Number:**

This protocol of updated network meta-analysis has been registered in Open Science Framework (OSF) system on January 8, 2021. The unique registration DOI number of 10.17605/OSF.IO/YSPM6 has been approved for our protocol (accessible at: https://osf.io/yspm6).

## 1 Introduction

Rheumatoid arthritis (RA) refers to a chronic autoimmune disease.^[1]^ The clinical manifest of RA primarily includes the following aspects: persistent synovitis, progressive joint injury, and deformity, which are the contributors to the disability and poor quality of life (QoL).^[2]^ Traditionally, methotrexate (MTX), as one of the conventional synthetic disease-modifying anti-rheumatic drugs (csDMARDs), is defined as the preferred therapy for the treatment of RA.^[3]^ Meanwhile, MTX is also regarded as the anchor drug for monotherapy or combination therapy with other drugs.^[4]^ For patients with moderate to severe active RA who is inadequately response to or who is intolerable to csDMARDs, combination therapy of csDMARDs or biologic DMARDs (bDMARDs) is preferentially recommended.^[5–7]^ Nevertheless, the extensive and long-lasting effects cannot be obtained in all patients with RA when csDMARDs and bDMARDs were prescribed for usage in clinical practice.^[8]^ Therefore, it is very important to develop novel alternative regimes for the treatment of RA.

Recently, the Janus kinases (JAKs) has been regarded as a promising target for treating RA^[9–11]^ because the expression of JAKs were detected to be increase in the synovium of patients with RA, as well as, target substrates, signal transducers and activators of transcription (STATs) which were all associated with the expression of JAKs were all identified.^[12–14]^ To date, several JAK inhibitors including tofacitinib, baricitinib, upadacitinib, filgotinib, and peficitinib have been approved to treat RA, and a Bayesian network meta-analysis performed by Lee and Song^[15]^ revealed that peficitinib may be the best treatment for achieving the American College of Rheumatology 20% (ACR20) response rate according to the ranking probability based on the surface under the cumulative ranking curve. Meanwhile, another systematic review and meta-analysis also indicated that no significant difference was found in cardiovascular risk among all JAKs inhibitors in a short-term perspective.^[16]^

It is noted that, moreover, a systematic review performed in China also revealed that 100 mg or 150 mg peficitinib may be the promising option for treating RA due to mild and tolerable adverse events (AEs).^[17]^ Considering a fact that different doses of peficitinib have been prescribed to treat RA in clinical practice, Lee and Song^[18]^ therefore performed another a Bayesian network meta-analysis of 3 randomized controlled trials to determine the comparative efficacy and safety of peficitinib 25, 50, 100, and 150 mg in treating active rheumatoid arthritis, and found that peficitinib 50, 100, and 150 mg once daily was effective for the treatment of active RA. However, this network meta-analysis did not include all potentially eligible studies for estimating the comparative efficacy of different peficitinib regimes because 2 eligible studies were missed.^[19,20]^ Therefore, we designed the current updated network meta-analysis to further investigate the comparative efficacy of different peficitinib regimes for the purpose of determining the optimal dosage of peficitinib in treating RA.

## 2 Methods

We registered the protocol of the current updated network meta-analysis in the Open Science Framework (OSF) platform on January 8, 2021. The registration DOI number of the current study is 10.17605/OSF.IO/YSPM6 (accessible at: https://osf.io/yspm6). As a result, the protocol of our network meta-analysis has been funded by a protocol registry. We developed main framework of performing the current network meta-analysis in accordance with process suggested by the Cochrane Collaboration.^[21]^ Moreover, we will report our findings according to the recommendations from the preferred reporting items for systematic review and meta-analysis (PRISMA) extension statement for reporting of systematic reviews incorporating network meta-analyses of health care interventions^[22]^ when the overall review were completed. For the current protocol, we designed the framework in line with the recommendations from the preferred reporting items for systematic review and meta-analysis protocols (PRISMA-P) 2015 statement.^[23]^ No ethical approval and informed consent will be required because we will perform all statistical analyses based on published studies.

### 2.1 Identification of records

**Figure 1:**
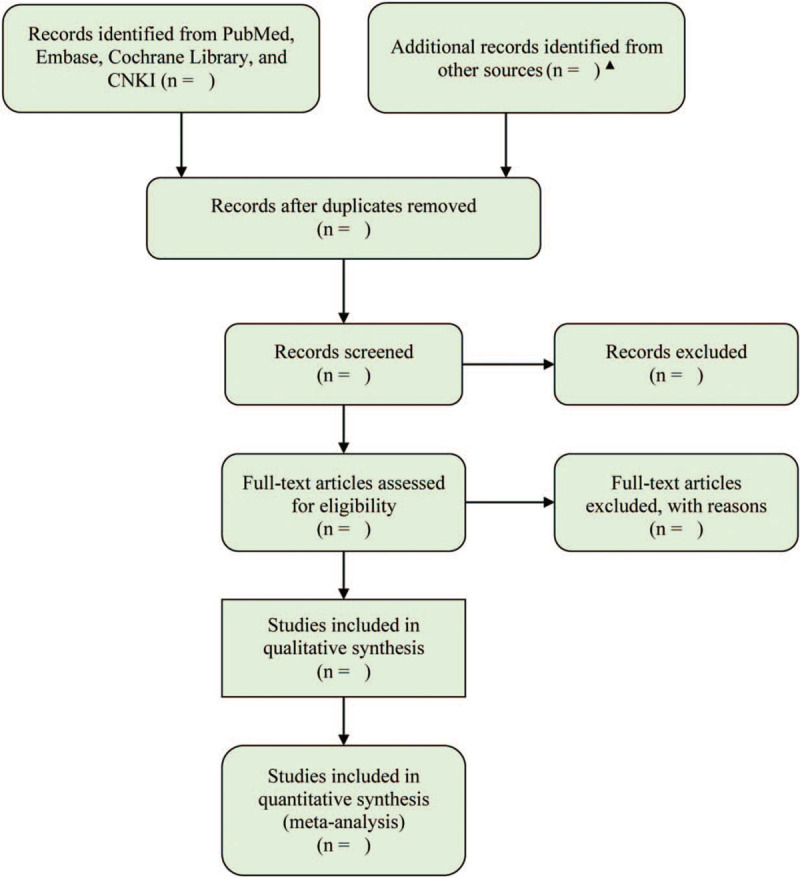
Flow diagram of identification and selection of eligible studies. ^▴^Additional records will be identified from the references of included studies and topic-related reviews.

We will perform a systematic search in PubMed, Embase, Cochrane Library, and China National Knowledgement Infrastructure (CNKI) in order to identify all available studies investigated the comparative efficiency of peficitinib and placebo or different peficitinib regimes. The process of searching literature will be performed by 2 independent investigators. We will simultaneously use the Medical Subject Heading (MeSH) and context words to develop the search strategy. In our network meta-analysis, we will use the following keywords and subject terms to construct search strings: “peficitinib” and “rheumatoid arthritis.” Meanwhile, we will also check the references of all included studies and topic-related reviews in order to add additional studies. The process of identification of studies was depicted in Fig. 1. Any disagreements about electronic search will be solved by consulting a third senior investigator.

### 2.2 Selection criteria

According to the previous network meta-analysis,^[18]^ we developed the following selection criteria: all adult patients were identified as RA according to the recognized criteria including the ACR criteria for RA^[24]^ or the classification criteria released jointly by ACR and European League Against Rheumatism (EULAR)^[25]^; peficitinib with or without csDMARD which was compared with placebo was prescribed to treat active RA; endpoints for the clinical efficacy and safety were reported; and the study design was randomized controlled trial. One study will be considered to be eligible for inclusion if it met the criteria as described above.

We will exclude review, case report, case series, and observational studies including quasi-experimental research, cohort study, case-control study, and cross-sectional study. We will also exclude studies without sufficient data if additional information could not be obtained after contacting corresponding author. About duplicate records, we will exclude one which was presented previously or has insufficient information. Any disagreements will be solved by consulting a third senior investigator during this stage.

### 2.3 Outcomes of interesting

In the current updated network meta-analysis, the number of patients who achieved an ACR20 response rate will be defined as the primary outcome of interesting. The number of patients who achieved ACR50 or ACR70 response rates and the number of patients withdrawn due to AEs will be considered as the secondary outcome of interesting.

### 2.4 Data extraction

We will assign 2 independent investigators to extract the following information from each included study: the name of the first author, year of publication, country in which the study was performed, details of treatment and control regimes, outcomes of interesting, and the details of risk of bias. At this stage, a standard information extraction sheet will be designed and then applied. Any disagreements at this stage will be resolved by consulting a third senior investigator.

### 2.5 Quality assessment

The risk of bias of an individual study will be assessed by using the Cochrane risk of bias assessment tool^[26]^ from the following 6 domains^[27]^: randomization, allocation concealment, blinding, incomplete data, selective outcome reporting, and other bias sources. Individual study will be labeled as “low,” “unclear,” or “high” risk of bias according to actual information. Any divergency will be resolved by consulting a third senior investigator. Eventually, we will grade the overall quality of each study to be low, moderate, or high quality according to the overall result of the risk of bias.

### 2.6 Statistical analysis

For conventional direct meta-analysis, we will calculate the odds ratio (OR) and corresponding 95% confidence intervals (CIs) to express binary outcomes based on the random-effect model because homogenesis is almost impossible across studies in the real world. We will firstly use the Cochran Q test to qualitatively evaluate the heterogeneity across studies,^[28]^ and then we will continue using *I*^2^ statistics to further quantitatively estimate heterogeneity.^[29]^ If the accumulated number of eligible studies was >10 for individual outcome, we will firstly draw funnel to qualitatively evaluate the possibility of presence of publication bias through inspecting the symmetry of the plot.^[30]^ And then, we will continue using Begg and Mazumdar adjusted-rank correlation test to quantitatively evaluate the publication bias.^[31]^ Review Manager 5.3 (version 5.3.5; Cochrane Collaboration, Copenhagen, Denmark) will be applied to perform direct meta-analysis.

After completing direct meta-analysis, we will conduct combination of direct and indirect evidences through performing Bayesian network meta-analysis by using the Markov Chain Monte Carlo methods, which was described by Lu and Ades.^[32,33]^ Network consistency will be assessed through evaluating the estimates between direct and indirect evidence using a node-splitting method.^[34]^ We will estimate the posterior distribution of all parameters using informative priors, and we will perform 50,000 simulation iterations and 20,000 burn-in iterations for the purpose of achieving convergence of the Markov Chain Monte Carlo model. Treatment regimes will be ranked in each iteration according to their outcomes been prepared on the basis of the surface under the cumulative ranking curve (SUCRA). The pooled results from network meta-analysis will be presented as odds ratio (OR) and the 95% credible interval (CrI). R software (version 3.2.3; R Foundation for Statistical Computing, Vienna, Austria) with GeMTC package (version 0.7-1; van Valkenhoef and Kuiper) will be used to perform network meta-analysis.

### 2.7 Sensitivity analysis

In order to assess the robustness of results from the network meta-analysis, we will also perform sensitivity analysis through excluding studies with a high-risk of bias and changing the model (fixed- and random-effects model) which is used to obtain pooled estimates.

### 2.8 Quality of evidence

In order to closely instruct decision making in clinical practice, we will use the Grading of Recommendations, Assessment, Development and Evaluations (GRADE) working group approach to rate the quality of evidence for results from the network meta-analysis.^[35]^ In this approach, the level of estimates from the direct evidence of RCTs was firstly defined to be high-quality, and the quality could be lowered to be moderate, low, and very low from 5 aspects including risk of bias, indirectness, imprecision, heterogeneity, and publication bias. For the indirect evidence, its quality will consistent with the lowest level of 2 direct comparisons which constructed the first-order loop of an indirect comparison. Certainly, quality of indirect evidence will also be lowered if imprecision or intransitivity was detected. Finally, the quality of estimates from network meta-analysis will be rated using the higher of the level between direct and indirect estimates if inconsistency between direct and indirect evidence was not identified.

## 3 Discussion

Rheumatoid arthritis is a common chronic and systematic autoimmune diseases, which is mainly characterized by synovitis and progressive joint destruction.^[1]^ Although csDMARDs and bDMARDs are the traditionally prescribed to treat RA, desirable treatment effects can not be obtained or remained in all patients with RA.^[2]^ Thus, researchers and practitioners have paid more attention to seek novel agent. As a non-receptor protein tyrosine kinases, JAK combines with transduction and activators of transcription (STATs) to primarily develop the signal transduction pathway which plays a critical role in immune responses, inflammatory reactions, and hematopoiesis.^[36]^ Several studies found that JAKs was increasingly expressed in the synovium of patients with RA, and their target substrates, signal transducers, and STATs were all also identified.^[2]^ Meanwhile, clinical studies and meta-analyses have also reported promising results when JAK inhibitors especially peficitinib were prescribed in treating RA.^[16,17,37,38]^ Although one meta-analysis has recently performed to investigate the comparative efficacy and safety of peficitinib 25, 50, 100, and 150 mg in patients with active RA, corresponding result must be cautiously interpreted because 2 eligible studies^[19,20]^ published in 2019 were not included. Therefore, it is imperative to further design an updated network meta-analysis to address this issue in order to generate more reliable evidence for clinical decision-making.

Regardless of a fact that our study will obtain more reliable and robust findings for decision-making through incorporating more adequate studies into network meta-analysis, some limitations should also be acknowledged. Firstly, our current meta-analysis will only search PubMed, Embase, Cochrane Library, and CNKI, however other electronic databases such as ISI Web of Science and Scopus will not be searched, which may cause inadequate identification of studies. Secondly, we will add additional 2 eligible studies in our meta-analysis compared with previous meta-analysis, however the accumulated number of eligible studies is still inadequate for obtaining greatly robust pooled results. Thirdly, we found all eligible studies only reported the follow-up results within 12 weeks, and therefore our meta-analysis will not obtain long-term effects of peficitinib in treating RA.

We have registered the protocol of our updated meta-analysis in the OSF system on January 8, 2021. The registration DOI number which has been approved for our protocol was 10.17605/OSF.IO/YSPM6 (accessible at: https://osf.io/yspm6). Currently, we have performed an initial search in targeted databases. Then, we will extract essential information before March 31, will calculate pooled estimates before 20 April, and will complete the full review before May 31, 2021.

On ethics approval and informed consent will not be required because our updated network meta-analysis will be performed based on published data. After completing the full review, we will disseminate findings through submitting it to the scholarly journal and conferences given filed.

## Acknowledgments

The authors must sincerely appreciate Open Science Framework (OSF) system because it develops an open access platform for registering study protocol.

## Author contributions

**Conceptualization:** Yuyi Zhou, Chunfang Sun, Chunyan Chen.

**Data curation:** Chunfang Sun.

**Investigation:** Yuyi Zhou.

**Methodology:** Yuyi Zhou.

**Validation:** Chunyan Chen.

**Visualization:** Chunyan Chen.

**Writing – original draft:** Yuyi Zhou, Chunfang Sun.

**Writing – review & editing:** Yuyi Zhou, Chunyan Chen.

## References

[1] Smolen JS Aletaha D McInnes IB. Rheumatoid arthritis. Lancet 2016;388:2023–38. 27156434 10.1016/S0140-6736(16)30173-8

[2] McInnes IB Schett G. Pathogenetic insights from the treatment of rheumatoid arthritis. Lancet 2017;389:2328–37. 28612747 10.1016/S0140-6736(17)31472-1

[3] Kameda H Fujii T Nakajima A. Japan College of Rheumatology guideline for the use of methotrexate in patients with rheumatoid arthritis. Mod Rheumatol 2019;29:31–40. 29718746 10.1080/14397595.2018.1472358

[4] Kay J Westhovens R. Methotrexate: the gold standard without standardisation. Ann Rheum Dis 2009;68:1081–2. 19525405 10.1136/ard.2008.102822

[5] O’Dell JR. Therapeutic strategies for rheumatoid arthritis. N Engl J Med 2004;350:2591–602. 15201416 10.1056/NEJMra040226

[6] Curtis JR Singh JA. Use of biologics in rheumatoid arthritis: current and emerging paradigms of care. Clin Ther 2011;33:679–707. 21704234 10.1016/j.clinthera.2011.05.044 PMC3707489

[7] Ciubotariu E Gabay C Finckh A. Joint damage progression in patients with rheumatoid arthritis in clinical remission: do biologics perform better than synthetic antirheumatic drugs? J Rheumatol 2014;41:1576–82. 25028383 10.3899/jrheum.130767

[8] Tanaka Y Izutsu H. Peficitinib for the treatment of rheumatoid arthritis: an overview from clinical trials. Expert Opin Pharmacother 2020;21:1015–25. 32345068 10.1080/14656566.2020.1739649

[9] Tanaka Y. Recent progress and perspective in JAK inhibitors for rheumatoid arthritis: from bench to bedside. J Biochem 2015;158:173–9. 26152731 10.1093/jb/mvv069

[10] Nakayamada S Kubo S Iwata S. Chemical JAK inhibitors for the treatment of rheumatoid arthritis. Expert Opin Pharmacother 2016;17:2215–25. 27690665 10.1080/14656566.2016.1241237

[11] Tanaka Y. The JAK inhibitors: do they bring a paradigm shift for the management of rheumatic diseases? Rheumatology (Oxford) 2019;58: (suppl): i1–3. 30806705 10.1093/rheumatology/key280 PMC6390877

[12] van der Pouw Kraan TC van Gaalen FA Kasperkovitz PV. Rheumatoid arthritis is a heterogeneous disease: evidence for differences in the activation of the STAT-1 pathway between rheumatoid tissues. Arthritis Rheum 2003;48:2132–45. 12905466 10.1002/art.11096

[13] Walker JG Ahern MJ Coleman M. Expression of Jak3, STAT1, STAT4, and STAT6 in inflammatory arthritis: unique Jak3 and STAT4 expression in dendritic cells in seropositive rheumatoid arthritis. Ann Rheum Dis 2006;65:149–56. 16096332 10.1136/ard.2005.037929 PMC1798020

[14] Isomäki P Junttila I Vidqvist KL. The activity of JAK-STAT pathways in rheumatoid arthritis: constitutive activation of STAT3 correlates with interleukin 6 levels. Rheumatology (Oxford) 2015;54:1103–13. 25406356 10.1093/rheumatology/keu430

[15] Lee HY Song GG. Comparative efficacy and safety of tofacitinib, baricitinib, upadacitinib, filgotinib and peficitinib as monotherapy for active rheumatoid arthritis. J Clin Pharm Ther 2020;45:674–81. 32495356 10.1111/jcpt.13142

[16] Xie W Huang Y Xiao S. Impact of Janus kinase inhibitors on risk of cardiovascular events in patients with rheumatoid arthritis: systematic review and meta-analysis of randomised controlled trials. Ann Rheum Dis 2019;78:1048–54. 31088790 10.1136/annrheumdis-2018-214846

[17] Liu X Xu CJ Zhong XY. Efficacy and safety of peficitinib for treating rheumatoid arthritis: a systematic review. China Pharm 2020;31:859–64.

[18] Lee YH Song GG. Comparative efficacy and safety of Peficitinib 25, 50, 100, and 150 mg in patients with active rheumatoid arthritis: a bayesian network meta-analysis of randomized controlled trials. Clin Drug Investig 2020;40:65–72. 10.1007/s40261-019-00863-9 31602572

[19] Takeuchi T Tanaka Y Tanaka S. Efficacy and safety of peficitinib (ASP015K) in patients with rheumatoid arthritis and an inadequate response to methotrexate: results of a phase III randomised, double-blind, placebo-controlled trial (RAJ4) in Japan. Ann Rheum Dis 2019;78:1305–19. 31350269 10.1136/annrheumdis-2019-215164 PMC6788880

[20] Tanaka Y Takeuchi T Tanaka S. Efficacy and safety of peficitinib (ASP015K) in patients with rheumatoid arthritis and an inadequate response to conventional DMARDs: a randomised, double-blind, placebo-controlled phase III trial (RAJ3). Ann Rheum Dis 2019;78:1320–32. 31350270 10.1136/annrheumdis-2019-215163 PMC6788921

[21] The Cochrane Collaboration, Higgins JPT Green S. Cochrane Handbook for Systematic Reviews of Interventions Version 5.1.0 [updated March 2011]. 2011.

[22] Hutton B Salanti G Caldwell DM. The PRISMA extension statement for reporting of systematic reviews incorporating network meta-analyses of health care interventions: checklist and explanations. Ann Intern Med 2015;162:777–84. 26030634 10.7326/M14-2385

[23] Moher D Shamseer L Clarke M. Preferred reporting items for systematic review and meta-analysis protocols (PRISMA-P) 2015 statement. System Rev 2015;4:1. 25554246 10.1186/2046-4053-4-1 PMC4320440

[24] Hochberg MC Chang RW Dwosh I. The American College of Rheumatology 1991 revised criteria for the classification of global functional status in rheumatoid arthritis. Arthritis Rheum 1992;35:498–502. 1575785 10.1002/art.1780350502

[25] Aletaha D Landewe R Karonitsch T. Reporting disease activity in clinical trials of patients with rheumatoid arthritis: EULAR/ACR collaborative recommendations. Arthritis Rheum 2008;59:1371–7. 18821648 10.1002/art.24123

[26] Higgins JP Altman DG Gotzsche PC. The Cochrane Collaboration's tool for assessing risk of bias in randomised trials. BMJ 2011;343:d5928. 22008217 10.1136/bmj.d5928 PMC3196245

[27] Higgins JP Green S. Cochrane handbook for systematic reviews of interventions version 5.0.0. Naunyn-Schmiedebergs Archiv 2008;5:S38.

[28] Higgins JP Thompson SG Deeks JJ. Measuring inconsistency in meta-analyses. BMJ 2003;327:557–60. 12958120 10.1136/bmj.327.7414.557 PMC192859

[29] Higgins JP Thompson SG. Quantifying heterogeneity in a meta-analysis. Stat Med 2002;21:1539–58. 12111919 10.1002/sim.1186

[30] Easterbrook PJ Berlin JA Gopalan R. Publication bias in clinical research. Lancet 1991;337:867–72. 1672966 10.1016/0140-6736(91)90201-y

[31] Begg CB Mazumdar M. Operating characteristics of a rank correlation test for publication bias. Biometrics 1994;50:1088–101. 7786990

[32] Lu G Ades AE. Combination of direct and indirect evidence in mixed treatment comparisons. Stat Med 2004;23:3105–24. 15449338 10.1002/sim.1875

[33] Dias S Sutton AJ Ades AE. Evidence synthesis for decision making 2: a generalized linear modeling framework for pairwise and network meta-analysis of randomized controlled trials. Med Decis Making 2013;33:607–17. 23104435 10.1177/0272989X12458724 PMC3704203

[34] van Valkenhoef G Dias S Ades AE. Automated generation of node-splitting models for assessment of inconsistency in network meta-analysis. Res Synth Methods 2016;7:80–93. 26461181 10.1002/jrsm.1167 PMC5057346

[35] Puhan MA Schünemann HJ Murad MH. A GRADE Working Group approach for rating the quality of treatment effect estimates from network meta-analysis. BMJ 2014;349:g5630. 25252733 10.1136/bmj.g5630

[36] Ghoreschi K Laurence A O'Shea JJ. Janus kinases in immune cell signaling. Immunol Rev 2009;228:273–87. 19290934 10.1111/j.1600-065X.2008.00754.x PMC2782696

[37] Genovese MC Greenwald M Codding C. Peficitinib, a JAK Inhibitor, in Combination with limited conventional synthetic disease-modifying antirheumatic drugs in the treatment of moderate-to-severe rheumatoid arthritis. Arthritis Rheumatol 2017;69:932–42. 28118538 10.1002/art.40054

[38] Kivitz AJ Gutierrez-Ureña SR Poiley J. Peficitinib, a JAK inhibitor, in the treatment of moderate-to-severe rheumatoid arthritis in patients with an inadequate response to methotrexate. Arthritis Rheumatol 2017;69:709–19. 27748083 10.1002/art.39955

